# Not brushing teeth at night may increase the risk of cardiovascular disease

**DOI:** 10.1038/s41598-023-37738-1

**Published:** 2023-06-28

**Authors:** Emiko Tanaka Isomura, Shinichiro Suna, Hiroyuki Kurakami, Shungo Hikoso, Toshihiro Uchihashi, Yusuke Yokota, Yasushi Sakata, Susumu Tanaka

**Affiliations:** 1 Department of Oral and Maxillofacial Surgery, Graduate School of Dentistry, Osaka University, 1-8 Yamadaoka, Suita, Osaka 565-0871 Japan; 2 Unit of Dentistry, Osaka University Hospital, Suita, Japan; 3 Department of Cardiovascular Medicine, Graduate School of Medicine, Osaka University, Suita, Japan; 4 Department of Medical Innovation, Osaka University Hospital, Suita, Japan

**Keywords:** Cardiology, Diseases, Health care, Medical research, Risk factors

## Abstract

In this study, we investigated whether toothbrushing timing affects cardiovascular disease risk. We enrolled 1675 patients aged ≥ 20 years who were hospitalized for surgery, examination, or medical treatment. The participants were categorized as follows based on toothbrushing: Group MN (brushing teeth after waking up and at night, n = 409), Group Night (brushing teeth at night but not upon waking up, n = 751), Group M (brushing teeth after waking up but not at night, n = 164), and Group None (not brushing teeth at all, n = 259). The participants’ age, sex, smoking history, and follow-up results were evaluated. Group M had four times as many men as women. Multivariate analysis of cardiovascular events showed significantly higher survival estimates in Group MN (*P* = 0.021) and Group Night (*P* = 0.004) than in Group None. Kaplan–Meier analysis of subgroups based on smoking status revealed that smokers in Group None had significantly worse prognosis for cardiovascular onset events than smokers in other groups; non-smokers in Groups None and M showed significantly worse prognosis on hospitalization. Our findings are limited to cardiovascular diseases and cannot be generalized to healthy populations. However, we suggest that brushing teeth at night is important for lowering cardiovascular disease risk.

## Introduction

In recent years, the importance of perioperative oral management has increased and is recommended for patients with malignancies, pulmonary diseases, gastrointestinal diseases, and cardiovascular diseases^1–3^. The association between oral management and malignancies, pulmonary diseases, and gastrointestinal diseases has been extensively reported, with the consensus that patients benefit from perioperative oral care^4,5^.

Dental diseases have been associated with cardiovascular disease risk. Several reports suggest that perioperative oral care positively impacts cardiovascular surgery outcomes^1,3,6–10^. Contrastingly, some reports suggest that oral hygiene is not associated with long-term prognosis in cardiovascular diseases and that people brushing their teeth more than twice a day have a greater risk of developing cardiovascular diseases than those brushing their teeth only once^2,11^.

Although “brushing teeth once a day” can imply brushing teeth at night after supper or before bedtime, we encounter many middle-aged and older patients in routine clinical practice who do not brush their teeth at night but only in the morning before breakfast. Many of them perceive the mouth to be unclean in the morning and thus brush their teeth before breakfast to avoid ingesting intraoral deposits. However, having breakfast also leads to intraoral deposits, which persist throughout the day and increase the risk of periodontal disease and dental caries.

Based on our clinical experience, patients not brushing their teeth at night have one of the following backgrounds: patients (1) may consume alcohol at night and go to bed without brushing their teeth or become too lazy to brush their teeth or (2) may be too tired after work and do not have adequate time to brush their teeth. On interviewing these patients, background (1) was found to be overwhelmingly common. Based on the perspective that intraoral plaque and deposits are present throughout the day, brushing only in the morning implies poor oral hygiene. We wondered if it also affected systemic diseases apart from periodontal disease and caries.

Reports on the timing of toothbrushing have mainly focused on its relationship with demineralization of the teeth^12–14^. To our knowledge, there are no reports on the relationship between toothbrushing timing and systemic diseases, including cardiovascular diseases. Therefore, we investigated whether the timing of toothbrushing affects the risk of cardiovascular diseases.

## Methods

### Study design and population

We enrolled 1,675 patients aged ≥ 20 years who were hospitalized at the Osaka University Hospital between April 2013 and March 2016 and visited its Unit of Dentistry during hospitalization. Ninety-two edentulous patients were excluded, resulting in a total of 1,583 patients. The purpose of hospitalization varied widely, from surgery to examination and medical treatment in departments. The reasons for visiting the Unit of Dentistry included preoperative oral care, screening for sources of infection, screening before steroid treatment, and requests for oral care or dental treatment. The study protocol was approved by the Clinical Ethics Committee of Osaka University Graduate School of Medicine, Osaka (approval number: 15044) and was conducted in compliance with the World Medical Association Declaration of Helsinki on medical research. The need for informed consent was waived by the same ethics committee (the Clinical Ethics Committee of Osaka University Graduate School of Medicine, Osaka). The participants were provided an opportunity to opt out on the hospital website and through posters posted in the dental treatment room, and patients who opted out of participation were excluded from the analysis.

### Measures

Dental and medical records of the participants were retrospectively reviewed by E.T.I., S.S., T.U., and Y.Y. To assess oral health, the pre-hospitalization frequency and timing of self-brushing were investigated through interviews, and the depth of periodontal pockets, degree of tooth mobility, and number of teeth were assessed by a single dentist (E.T.I). The depth of the periodontal pocket was measured by a dental hygienist by inserting a dental probe in the gingival sulcus at six points (mesial, central, and distal points on both buccal and lingual sides of the tooth). To examine the degree of tooth mobility, the dental hygienist pinched the tooth with tweezers and moved it and measured the degree of sway; the progress of the periodontal disease was assessed based on the degree (Miller index: 0, no detectable movement on applying force; 1, greater than normal movement; 2, movement no greater than 1 mm in the buccolingual direction; and 3, movement of more than 1 mm in the buccolingual direction with depressibility)^15^. The timing of toothbrushing was categorized into five groups: after waking up (before breakfast), after breakfast, after lunch, after supper, and before bedtime. Group MN included participants who brushed their teeth after waking up and at night, Group Night included those who brushed their teeth at night but not upon waking up, Group M included those who brushed their teeth after waking up but not at night, and Group None included those who did not brush their teeth at all. Patients who brushed their teeth after supper or before bedtime were categorized in the same group, and the timing of brushing teeth after breakfast or lunch was not considered. Missing data were recorded as “unknown” and not included in the analyses, resulting in a reduced sample for some analyses.

The patients’ medical information (including age; sex; smoking history; serum levels of C-reactive protein [CRP], hemoglobin, albumin, hemoglobin A1c [HbA1c], and brain natriuretic peptide [BNP] at admission; and follow-up observation results) was examined. The presence or absence of cardiovascular events, presence or absence of hospitalization, and life prognosis (regardless of the cause of death) were investigated based on electronic medical records. The observation period was until death or the end of the observation period (June 30, 2016). Cardiovascular events were defined as hospitalization for cardiovascular diseases, including the composite of heart failure, myocardial infarction, arrhythmia, angina pectoris requiring coronary revascularization, valvular diseases requiring surgery, and aortic diseases requiring surgery.

### Statistical analyses

An exploratory analysis was conducted by univariate and multivariate analyses of the proportional hazards model for the relationship between the observation items and the occurrence of events such as cardiovascular events or life prognosis. In addition, for subgroups defined by smoking status, the Kaplan–Meier method was used to estimate the time from the participants’ visit to the Unit of Dentistry to the occurrence of the endpoint, and the log-rank test was performed. All statistical analyses were performed using SAS version 9.4 (SAS Institute, Cary, NC, USA). *P*-values < 0.05 were considered to indicate significant differences.

## Results

There were 1583 target patients. Group MN (brushed their teeth in the morning and at night) had 409 patients (25.8%), Group M (brushed their teeth only in the morning) had 164 patients (10.4%), Group Night (brushed their teeth only at night) had 751 patients (47.4%), and Group None (did not brush their teeth at all) had 259 patients (16.4%). Group Night had the highest percentage of brushing after lunch (44.9%), followed by Group MN (24.0%), while almost no patients in Groups M and None brushed after lunch. The detailed characteristics of each group are presented in Table 1.

**Table 1:** Participant characteristics.

		Group MN	Group night	Group M	Group none
		(n = 409)	(n = 751)	(n = 164)	(n = 259)
Age (years)	Median (min, max)	65.00 (20.00, 95.00)	66.00 (20.00, 97.00)	68.00 (20.00, 89.00)	63.00 (22.00, 94.00)
Sex	Male (%)	224 (54.9)	412 (54.9)	132 (80.5)	153 (59.1)
	Female (%)	185 (45.2)	339 (45.1)	32 (19.5)	106 (40.9)
Brushing after lunch	Yes (%)	98 (24.0)	337 (44.9)	5 (3.0)	17 (6.6)
	No (%)	311 (76.0)	414 (55,1)	159 (97.0)	242 (93.4)
CRP at admission	Available data	407	748	164	217
(mg/dl)	Median (min, max)	0.14 (0.02, 17.67)	0.16 (0.02, 32.86)	0.18 (0.02, 12.66)	0.24 (0.02, 17.51)
Hemoglobin at admission	Available data	408	750	164	218
(× 10^6^/μl)	Median (min, max)	12.60 (5.70, 17.70)	12.40 (3.00, 18.00)	12.80 (6.70, 18.80)	12.70 (4.60, 17.90)
Albumin at admission	Available data	408	748	164	218
(g/dl)	Median (min, max)	3.90 (0.80, 4.90)	3.90 (0.90, 5.30)	3.90 (1.50. 5.10)	3.80 (1.40, 5,00)
Creatinine at admission	Available data	409	751	164	218
(mg/dl)	Median (min, max)	0.80 (0.33, 9.87)	0.77 (0.25, 15.92)	0.91 (0.48, 7.89)	0.81 (0.30, 11.53)
HbA1c at admission	Available data	158	292	65	94
(%)	Median (min, max)	5.30 (3.30, 15.10)	5.50 (3.20, 11.00)	5.40 (3.80, 16.50)	5.50 (3.30, 9.50)
BNP at admission	Available data	251	465	97	142
(mg/dl)	Median (min, max)	72.20 (2.00, 2875.90)	66.10 (2.00, 4400.10)	52.30 (2.00, 1665.30)	88.85 (2.00, 9157.90)
Smoking	Yes (%)	68 (16.9)	118 (16.0)	35 (21.9)	33 (22.9)
	No (%)	355 (83.1)	620 (84.0)	125 (78.1)	111 (77.1)
Gingival pocket depth	More than 1 pocket over 8 mm (%)	104 (25.4)	149 (19.9)	29 (17.7)	37 (18.2)
	None over 8 mm (%)	305 (74.6)	601 (80.1)	1135 (82.3)	166 (81.8)
Tooth mobility*	More than 1 tooth with mobility 3 (%)	45 (11.0)	71 (9.5)	16 (9.8)	27 (13.8)
	No tooth with mobility 3 (%)	364 (89.0)	677 (90.5)	148 (90.2)	175 (86.7)

CRP, C-reactive protein; HbA1c, hemoglobin A1c; BNP, brain natriuretic peptide; SD, standard deviation; Group MN, brushing in the morning and at night; Group M, brushing only in the morning; Group Night, brushing only at night; Group None, no brushing at all.

*Tooth mobility (Miller index).

0: no detectable movement when force was applied.

1: greater than normal movement (physiological).

2: movement no greater than 1 mm in the buccolingual direction.

3: movement of more than 1 mm in the buccolingual direction with depressibility.

There was no age-based difference among the groups; not only middle-aged and older patients but also some young patients did not brush their teeth. Further, there were four times more men than women in Group M. Blood examination was not performed at the time of admission in some cases, and participants without blood reports were excluded. The number of participants with available data on the following parameters were as follows: CRP level, 1536; hemoglobin level, 1540; albumin level, 1538; creatinine level, 1542; HbA1c level, 609; and BNP level, 955. There were no differences in parameters, except for BNP level, among the groups. There were 5.5–7.7% more patients with pocket depths > 8 mm in Group MN than in the other three groups. There were more patients with a mobility index of 3 in Groups None and MN than in Groups Night and M. Smoking was equally common in all groups.

Univariate and multivariate analyses of cardiovascular events showed significantly higher survival estimates in Groups MN (*P* = 0.021) and Night (*P* = 0.004) than in Group None (Table 2). There was a significant difference in BNP data at the time of admission in the univariate analysis, but BNP data were excluded from the multivariate analysis because only 955 participants had BNP data at the time of admission of all 1583 participants, which is only 60% of the total. There were also significant differences in age, serum levels of albumin and creatinine, and smoking status. However, on univariate and multivariate analyses of life prognosis, only hemoglobin level at admission was significantly different (Table 3).

**Table 2:** Univariate and multivariate Cox proportional hazard models of cardiovascular events.

		Univariate model			Multivariate model (n = 1439)	
		n	Hazard ratio [95% CI]	*P* value	Hazard ratio [95% CI]	*P* value
Timing of brushing	MN	1583	0.795 [0.535–1.179]	0.254	0.591 [0.378–0.923]	0.021*
	Night		0.757 [0.531–1.079]	0.124	0.550 [0.365–0.830]	0.004*
	M		0.879 [0.542–1.428]	0.603	0.633 [0.374–1.072]	0.633
	None (ref)		–	–	–	–
Age		1583	1.011 [1.002–1.020]	0.013*	1.019 [1.009–1.029]	< 0.001**
Sex	Male	1583	1.013 [0.777–1.319]	0.927		
	Female (ref)		–	–		
CRP at admission		1536	0.911 [0.849–0.978]	0.010*	0.940 [0.867–1.018]	0.130
Hemoglobin at admission		1540	1.030 [0.971–1.093]	0.324		
Albumin at admission		1538	1.775 [1.393–2.261]	< 0.001**	1.885 [1.410–2.521]	< 0.001**
Creatinine at admission		1542	1.115 [1.030–1.208]	0.007*	1.166 [1.067–1.274]	< 0.001**
HbA1c at admission		609	1.014 [0.856–1.200]	0.875		
BNP at admission		955	1.000 [1.000–1.000]	< 0.001**		
Gingival pocket depth	More than 1 pocket over 8 mm	1526	1.191 [0.875–1.621]	0.265		
	None over 8 mm (ref)		–	–		
Tooth mobility	More than 1 tooth with mobility 3	1524	0.832 [0.520–1.331]	0.442		
	No tooth with mobility 3 (ref)		–	–		
Smoking	Yes	1445	0.590 [0.386–0.903]	0.015*	0.596 [0.389–0.914]	0.018*
	No (ref)		–	–	–	–

CRP, C-reactive protein; HbA1c, hemoglobin A1c; BNP, brain natriuretic peptide; CI, confidence interval; Group MN, brushing in the morning and at night; Group M, brushing only in the morning; Group Night, brushing only at night; Group None, no brushing at all.

*P < 0.05, ** *P* < 0.001.

**Table 3:** Univariate and multivariate Cox proportional hazard models of life prognosis.

		Univariate model			Multivariate model (n = 1533)	
		n	Hazard ratio [95% CI]	*P* value	Hazard ratio [95% CI]	*P* value
Timing of brushing	MN	1583	1.231 [0.754–2.009]	0.406	1.069 [0.654–1.749]	0.789
	Night		1.243 [0.792–1.951]	0.345	1.050 [0.667–1.653]	0.832
	M		0.901 [0.473–1.718]	0.752	0.862 [0.450–1.652]	0.655
	None (ref)		–	–	–	–
Age		1583	1.008 [0.998–1.018]	0.117		
Sex	Male	1583	1.216 [0.897–1.649]	0.208		
	Female (ref)		–	–		
CRP at admission		1536	1.069 [1.035–1.104]	< 0.001**	1.037 [0.998–1.077]	0.065
Hemoglobin at admission		1540	0.827 [0.778–0.880]	< .0001**	0.857 [0.801–0.916]	< 0.001**
Albumin at admission		1538	0.618 [0.505–0.756]	< 0.001**	0.783 [0.612–1.003]	0.052
Creatinine at admission		1542	1.043 [0.927–1.174]	0.484		
HbA1c at admission		609	0.865 [0.696–1.075]	0.190		
BNP at admission		955	1.000 [1.000–1.000]	0.691		
Gingival pocket depth	More than 1 pocket over 8 mm	1526	0.963 [0.669–1.385]	0.838		
	None over 8 mm (ref)		–	–		
Tooth mobility	More than 1 tooth with mobility 3	1524	0.939 [0.561–1.571]	0.810		
	No tooth with mobility 3 (ref)		–	–		
Smoking	Yes	1445	1.021 [0.683–1.524]	0.921		
	No (ref)		–	–	–	–

CRP, C-reactive protein; HbA1c, hemoglobin A1c; BNP, brain natriuretic peptide; CI, confidence interval; Group MN, brushing in the morning and at night; Group M, brushing only in the morning; Group Night, brushing only at night; Group None, no brushing at all.

**P < 0.001.

**Figure 1:**
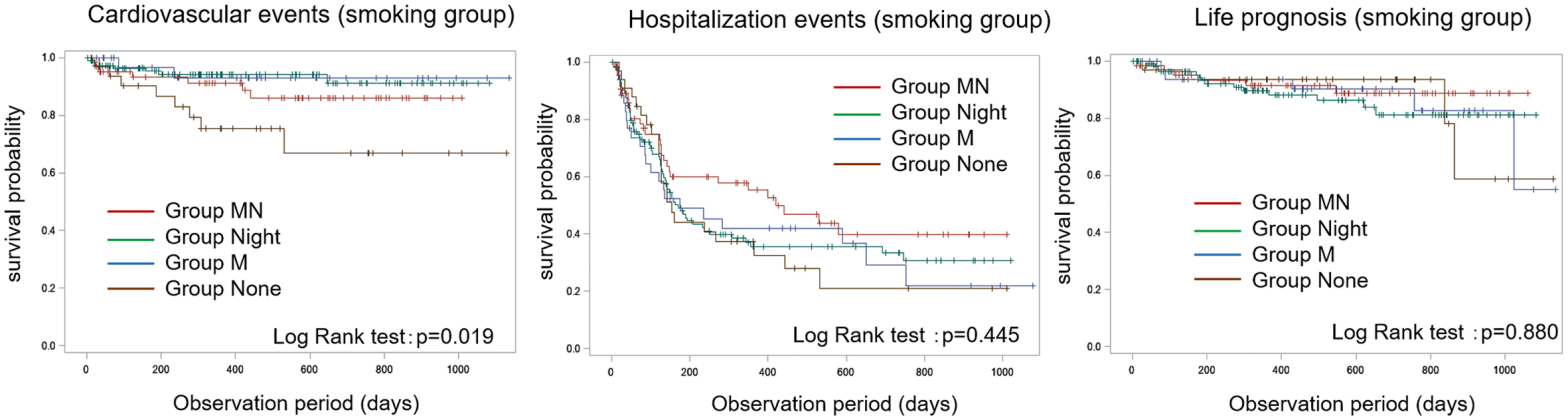
Kaplan–Meier analysis plots for cardiovascular events, hospitalization events, and life prognosis among smokers. Group None had a significantly worse prognosis for cardiovascular onset events with smoking than the other groups.

**Figure 2:**
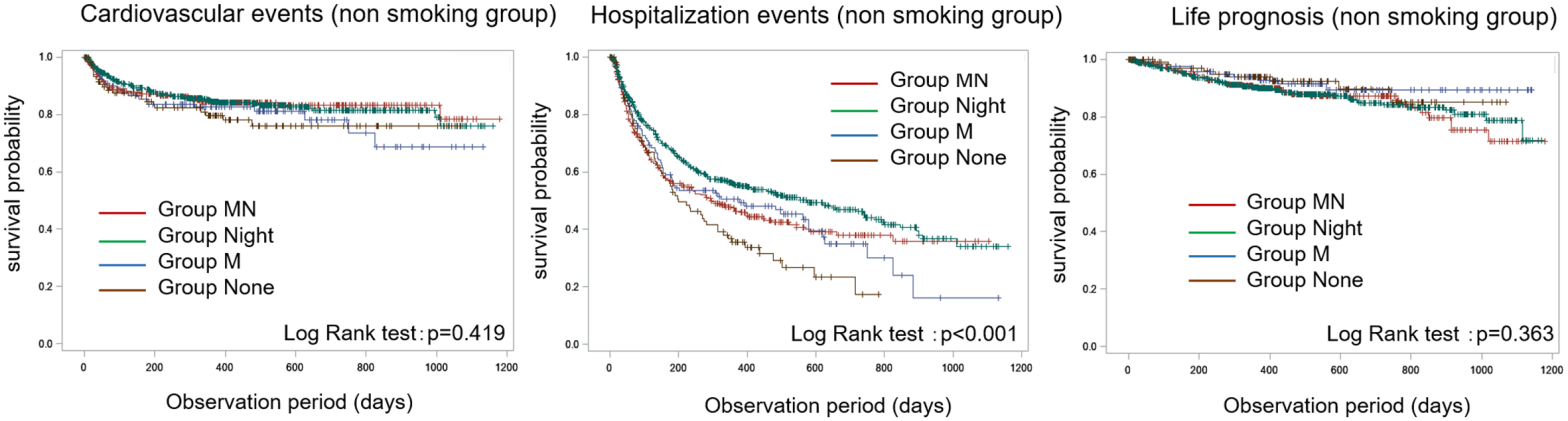
Kaplan–Meier analysis plots for cardiovascular events, hospitalization events, and life prognosis for non-smokers. Non-smoking participants with hospitalization events in Group None and Group M showed a worse prognosis.

Kaplan–Meier analysis of subgroups based on smoking status revealed that smokers in Group None had worse prognosis for cardiovascular onset events than those in the other groups. Non-smoking participants with hospitalization events in Groups None and M showed worse prognosis (Figs. 1 and 2).

## Discussion

In this study, we investigated cardiovascular events and life prognosis in relation to the timing of toothbrushing. Although we encounter many middle-aged and older patients who do not brush their teeth at night in clinical practice, the present findings indicate that there was a certain percentage of individuals not brushing their teeth at night in each age group. This may be attributable to the effect of parental habits. Some people were taught in childhood to brush their teeth after waking up (before breakfast), regardless of whether teeth were brushed at night. Toothbrushing habits may also be associated with lifestyle and regional differences. Participants who only brushed in the morning and those who did not brush at all demonstrated a tendency to not brush after lunch either, suggesting a lack of interest in dental hygiene. Thus, we did not create a separate group for people who did not brush after lunch because this population was almost identical to the population who did not brush at night in this study.

Since this study was limited to hospitalized patients, their poor physical condition may have impeded oral health maintenance and affected their prognosis. In addition, as this is a retrospective study, there may have been selection bias. Since only inpatients at one hospital were included, the skills and treatment strategies of the attending physicians may not be the same as those at other hospitals. Since ours is a university hospital, it manages many difficult cases and is biased in the type of cases it handles. While cardiac disease is generally thought to be unique to older people, many of our patients had severe congenital heart disease and some had artificial assistive hearts or died while waiting for heart transplants. In other words, not all young patients at our hospital are healthy. Therefore, we included patients aged 20 years and older. A multicenter study is needed to generalize the results of this study. In addition, the median observation period for the 1583 patients was 441 days, during which time 176 deaths, 226 cardiovascular events, and 762 hospitalizations occurred. The short cohort period is also a limitation of this study.

However, the findings clearly indicate that only brushing in the morning after waking up is inadequate and that brushing at night is good to maintain good health. These implications are consistent with the theory that the intraoral bacterial load increases during sleep at night due to reduced salivary flow^16,17^.

Although the complete mechanism is unknown, the following mechanisms have been suggested: (1) the loss of teeth due to periodontal disease or dental caries due to an increase in oral bacteria impairs chewing efficiency and health; (2) disruption of the balance of intestinal bacteria by oral bacteria can impair health^18,19^; and (3) cardiovascular events develop from bacteremia caused by periodontal disease.

There were no significant differences in cardiovascular events, hospitalization events, or life prognosis among the groups based on the number of teeth, caries, or periodontal disease. A lower number of teeth is associated with lower chewing strength. Tanaka et al.^20^ included a low number of remaining teeth as a factor of oral frailty, which exacerbates physical frailty, sarcopenia, and mortality, but they stated that there was no significant difference in health deterioration based on the number of teeth alone. Retention of teeth with advanced periodontal disease creates a risk of endocarditis. In our department, we encourage patients with cardiovascular diseases to actively undergo extraction of teeth with endodontic or periodontal issues and poor prognosis. In fact, Lockhart et al.^17^ reported that bacteremia after toothbrushing was associated with poor oral hygiene and gingival bleeding after toothbrushing.

In the present study, it is unclear if the participants brushed their teeth properly because they were not judged based on objective measures of oral hygiene, such as the plaque index. In addition, data on auxiliary tools, such as floss and interdental brushes, and time spent brushing teeth were not included because they would complicate the analysis and would not directly lead to an evaluation of whether the patients were brushing their teeth adequately even if they spent more time doing so or used auxiliary tools. Therefore, the plaque score should also have been evaluated. We did not include it in the analysis because only a part of the sample underwent plaque score examination. Further, many patients in Group MN had gingival pockets > 8 mm. A periodontal pocket of 8 mm was chosen as the cutoff value because pockets > 8 mm are indications for tooth extraction as they are a source of infection due to severe periodontal disease. However, it is unclear if the participants were more conscious regarding toothbrushing due to existing periodontal disease or that their teeth were preserved despite advanced periodontal disease because of their enthusiastic brushing. Patients with pockets deeper than 8 mm have advanced periodontal disease and may already be affected by cardiovascular disease, even if they belong to Group MN, which is a limitation of this study. However, although not included in the Kaplan–Meier analysis in this study, the presence or absence of cardiac events and life prognosis were compared after excluding those with pockets deeper than 8 mm, and there was almost no difference.

Although our findings are limited to cardiovascular diseases and cannot be applied to healthy individuals, they indicate that brushing teeth at night is important. To prevent cardiovascular diseases, brushing teeth before breakfast is necessary, but most important is brushing teeth at night before going to bed. The conventional oral hygiene routines followed in Japan may not be optimal for good systemic health. Therefore, it is necessary to increase awareness among the general population regarding the appropriate timing for brushing teeth.

## Data Availability

The datasets analyzed in the current study are available from the corresponding author on request.
